# Conceptualizing Community Oriented Primary Care (COPC) – the Tshwane, South Africa, health post model 

**DOI:** 10.4102/phcfm.v5i1.423

**Published:** 2013-02-22

**Authors:** Nomonde Bam, Tess Marcus, Jannie Hugo, Hans-Friedemann Kinkel

**Affiliations:** 1 Department of Family Medicine, University of Pretoria, South Africa

## Introduction

### Re-engineering primary health care

Health sector reforms initiated in South Africa in 1994 adopted a primary health care approach to strengthen the health system and achieve public health outcomes through disease prevention and health promotion.^1^ This led to the building of many new clinics to make health services more accessible, affordable and equitable. However, the pressures of a predominantly hospital-centric health care system and emerging epidemics prevented the successful provision of quality comprehensive, integrated primary care to millions of South Africans. Also, little attention was given to community partnerships and multi-sectoral collaboration.

This had two related negative consequences for health care services. Primary health care became a passive, low quality and episodic service of individuals in clinics, whilst hospitals were overburdened with referrals and patients seeking help. Compounded by a burgeoning burden of disease, pressure on the system increased exponentially and it was therefore not able to improve health outcomes.^2^

Dr Aaron Motsoaledi, the minister of health, intended to address these challenges in the 2010 ‘Revitalisation of Primary Health Care’ initiative by re-engineering primary health care towards proactive household- and community-focused interventions.^3^ Acknowledging health care as a universal right, the approach encompasses promotion of healthy living, disease prevention, early detection, treatment of illness, community-based disease management and rehabilitation. Practically, the initiative comprise of municipal ward outreach teams, district specialists teams and school health teams.^3,4^

Following the National Minister of Health’s initiative, the then member of the executive committee (MEC) for health in Gauteng, Ms Qedani Mahlangu, announced the adoption of Community Oriented Primary Care (COPC) as Gauteng’s approach to re-engineering primary health care. This was articulated in the 2010–2020 Service Transformation Plan and saw the creation of health posts.^5^ It then cascaded to the district level, where the University of Pretoria’s Faculty of Health Sciences, through its Community Engagement office, facilitated a process with the Tshwane District Office of the Gauteng Department of Health and Social Development. In 2010, the departments of Family Medicine at the Universities of Pretoria and Limpopo, together with the Tshwane District Office of the Gauteng Department of Health and Social Development, developed a conceptual framework for implementing COPC through health posts in Tshwane. The approach was supported by USAID funding through the Foundation for Professional Development (FPD).

This article briefly describes the health post model of COPC in Tshwane as an approach to primary health care re-engineering. How the model unfolds in the future will be described and analysed in subsequent publications based on a built-in process of participatory action research (The National Department of Health is using the term ‘Ward Based Outreach Team’ [WBOT] in place of ‘Health Post’ and that is the term that is used in the field and in future publications).

### Community Oriented Primary Care (COPC)

With the implementation of COPC, the Tshwane District Office of the Gauteng Department of Health joins a globally established approach to primary health care that originated in South Africa about seven decades ago with Sidney and Emily Kark.^6,7,8,9,10^ (The history of COPC in South Africa is summarised elsewhere^8,11,12^).

Briefly, COPC is grounded in the notion that people’s health is determined by their social environment.^13,14^ This means that individual and population level improvements in health cannot be achieved without simultaneously changing the social determinants that shape health more generally. COPC has been summarily described as ‘the merger of front line clinical medicine with public health’.^11,14^ As such, COPC addresses individual health needs in the collective context of family and community. COPC is characterised by local specificity that derives from the behavioural, cultural and social characteristics of people who live in particular places.^14^ Also, COPC is rendered by multi-disciplinary teams that work collaboratively with local people. COPC uses epidemiology as a tool for practicing health care and evaluating its effects on a systematic and continuous basis.^15,16,17^ It also integrates research, teaching and learning into the practice of health care delivery.^18,19,20^

COPC contributes to broader human development both by advancing health literacy and social capabilities, as well as by engaging with the socio-economic and politico-cultural determinants of health and disease.^21^ As such, health care is achieved through empowerment as much as through improved service delivery.^22,23,24^ 

### The Tshwane Health Post Model

COPC in Tshwane District,

[...] is designed around health posts. These are both structures that are physically located in communities and they are health care practitioner teams, initially comprising professional nurses and CHWs [community health workers]. As teams, their responsibility is to interact in a proactive way with every household (as a collective, as well as with individual members) in their jurisdiction. Their role is to promote health, prevent disease and detect disease early, and support treatment, rehabilitation and palliation, and to do this in a way that both develops capacity and shared responsibility for heath care between service providers and service users.^25^

### What the health post is

The health post is developed as a service unit that is physically located in a community and serves the population in a defined geographic area within a specific municipal ward.^5,26^ It has been conceptualised as a ‘nerve centre’ that drives and coordinates all primary care interventions in the community.^5,21^

Each health post serves between 2000 and 3000 households in a defined geographic area within a municipal ward.^5^ Whilst the intention is to expand health posts to the 44 wards of Tshwane District, the first nine health posts were initiated in the poorest wards in priority areas in each sub-district of the region. On initiation, the geophysical area served by the health post is identified and mapped using ward-based maps to define the boundaries of health post activity. Each community health worker (CHW) is assigned between 150 and 200 households, although the ideal ratio of households per CHW has yet to be tested in practice for feasibility and impact. 

At its core, the health post team comprises a health post manager and between 20 and 40 CHWs - proportionate to the number of households served. The health post manager is a professional nurse and the CHWs are recruited from the communities surrounding the health posts. This approach enables the team to work from a sound understanding of the local culture and idiom, although there is the risk of overfamiliarity and problems associated with privacy.^25^

For the most part, the health post is hosted by existing community-based nonprofit organisations (NPOs). This approach to COPC was deliberately adopted because these organisations have historically established structures and links in the communities. Their familiarity with local people and practices provides a base for COPC and shortens the time it takes to develop acceptability and mutual trust. In addition, their knowledge of the communities makes a significant contribution to planning and intervention. Lastly, they have administrative structures in place that facilitate employment relations and daily team functioning. The linking of the health posts to existing NPOs also inevitably poses some challenges to all parties, since they each bring their own practice legacies around focus of service, funding and authority structures.^25^

### What the health post does

As an entry point into the health and social care systems, health post teams engage with individuals and families in a continuous, comprehensive and integrative way that is information-led. Continuity means building interpersonal relations between individuals and families, and members of the health post team over time. Continuity also means continuity of care over the course of a condition or life period. Comprehensiveness means ensuring that the multiplicity of health needs, from promotion to palliation, are responded to in a reflective way. Integration means engaging with health-related socio-economic, cultural and environmental issues directly with individuals and families and building working relations with relevant partner organisations and institutions in the respective areas. Within the health care system, integration means collaborating actively with school health and district specialist teams in the envisaged National Health Insurance (NHI) model.

COPC begins by familiarising the health post team with the organisations that are active in their sites and the individuals and families who live in the households they will serve. Practically, the former involves conducting an institutional assessment to see how existing organisations work and the contribution they could make to the health post. The latter involves carrying out household registration and individual structured health status assessments in order to establish individual, household and community baselines. These are used to understand the predominant health issues and to determine priority interventions at all levels. This assessment is ongoing so that new issues can be identified and responded to as they arise in a continuous and iterative way.

The health status assessment data is captured electronically by the health post team using mobile phones. The data is transmitted to a web-based data platform. It is then kept at the health post as a family file. Using daily electronic reports on all CHW visits and interventions, as well as additional verbal reports from CHWs, the health post manager is able to allocate daily tasks and address health and social challenges for each household as they arise.^27^ The family files are also confirmed, refined and updated through regular home visits. In addition, data is aggregated for community level assessments and broad strategic health campaigns. The health post team, together with district managers, the University of Pretoria and other collaborating partners, use this data to determine short, medium and longer term interventions. By the end of July 2012, over 40 000 individual health status assessments had been carried out at the health posts and prioritization for strategic intervention was in progress.

## Conclusion

With its health post model, Tshwane District has embarked on an innovative journey to address the health needs of ordinary South Africans through COPC. It focuses on continuous, comprehensive, integrated and informed health care. As an active collaboration between health services, universities and funding agencies and communities, the Tshwane model creates a platform on which to successfully re-engineer primary health care in South Africa.
